# The Development of the Chinese Sentiment Lexicon for Internet

**DOI:** 10.3389/fpsyg.2019.02473

**Published:** 2019-11-05

**Authors:** Jia-Lin Zhao, Meng-Zhu Li, Juan Yao, Ge-Hua Qin

**Affiliations:** ^1^Department of Sociology, School of Philosophy, Law and Political Science, Shanghai Normal University, Shanghai, China; ^2^Finance Discipline, Business School, The University of Sydney, Darlington, NSW, Australia; ^3^The School of Electronic Information and Electrical Engineering, Shanghai Jiao Tong University, Shanghai, China

**Keywords:** Chinese sentiment lexicon, valence and arousal, online social media, reliability and validity, personality traits

## Abstract

This paper examines the development of the Chinese Sentiment Lexicon for Internet (CSLI), a sentiment lexicon for capturing the valence and arousal in Chinese online social media texts. We first review the current sentiment lexicons and their building process, including the collection of words, judging the emotionality of words, and testing reliability and validity. In Study 1, we develop CSLI and test its initial reliability and validity. In Study 2, we further test the convergent validity of CSLI by examining its correlations with human judgment in 429 aggregated Weibo comments. In Study 3, the predictive validity of CSLI is examined by linking its results to personality traits among 52 undergraduates. Two replication studies are also conducted to verify the findings in Study 2 and 3. The results have generally supported the reliability and validity of CSLI. Therefore, CSLI can be used as a research tool to capture the degree of valence and arousal in Chinese online social media texts. Its potential to promote human well-being is also discussed.

## Introduction

Sentiment analysis of texts in online social media (e.g., Facebook and Twitter) has been a field of research attracting much attention over the past decade (Chew and Eysenbach, 2010; Bollen et al., 2011; Thelwall et al., 2012). A major approach is to use a sentiment lexicon to automatically extract emotional information from those texts (e.g., counting the frequency of the “emotion” words in the lexicon; Calvo and Kim, 2013). Commonly used English sentiment lexicons include Financial Sentiment Dictionary (Loughran and McDonald, 2011), Google-Profile of Mood States (Bollen et al., 2011), Linguistic Inquiry and Word Count (Pennebaker et al., 2007), OpinionFinder (Wilson et al., 2005), SentiWordNet (Miller et al., 1990), and WordNet-Affect (Bobicev et al., 2010). There is evidence that emotions captured by those lexicons in online social media are associated with personality traits (Schwartz et al., 2013; Cutler and Kulis, 2018), and can predict sales results (Hu et al., 2014) and stock price changes (Bollen et al., 2011).

So far, research on sentiment analysis has been limited largely to English-language text. Lexicons in other languages, especially in Chinese, remain relatively rare. Yet nearly half of all internet users are from Asian countries such as China (Miniwatts Marketing Group, 2018). While there have been some studies on the development of a Chinese sentiment lexicon (e.g., Dong and Dong, 1999; Ku et al., 2006; Dong et al., 2015), compared to the English lexicons, there is ample scope and a pressing need to further the research in Chinese texts. First, there have been few Chinese lexicons designed for capturing texts in online social media featuring informal and newly invented words, such as 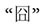 (embarrassed). The only published lexicon for this purpose appears to be that by Dong et al. (2015). Second, current Chinese lexicons such as HowNet sentiment (Dong and Dong, 1999) and National Taiwan University Sentiment Dictionary (Ku et al., 2006) can only output the dimension of valence (i.e., the degree of positivity/negativity) from the text, but not include other dimensions or categories of emotions (e.g., the dimension of arousal). Third, it is still necessary to further establish the reliability and validity of Chinese sentiment lexicons, particularly their predictive validity (i.e., predicting real life outcomes). The lexicon built by Dong et al. (2015), for instance, has only been examined with the fluctuation of emotions in several public events.

The main aim of the current research is to further develop a reliable and valid Chinese lexicon, suitable for capturing the emotionality in Chinese online social media (e.g., Weibo and WeChat). We also included both dimensions of valence and arousal as the output of the lexicon to provide more information on emotions. To serve the purpose, we first reviewed the building process of the current sentiment lexicons. Based on the review, we further developed the Chinese Sentiment Lexicon for Internet (CSLI) in Study 1. We then examined the construct validity of CSLI in Study 2 by comparing its outcomes with human judgment in Weibo comments. Finally, we tested the predictive validity of CSLI in Study 3 by linking it to the personality traits of undergraduate students. For Study 2 and 3, we also conducted *post hoc* replication studies to verify their results.

## The Building Process of Current Sentiment Lexicons

### The Collection of Words

Collecting words is generally the first step in building a sentiment lexicon. There are two major concerns: Where do the initial words come from? How are they selected/screened? For the first question, current sentiment lexicons are mainly built upon previous dictionaries or measurement of emotions. For instance, the initial words in the Linguistic Inquiry and Word Count were drawn from common emotion scales and standard English dictionaries (Pennebaker et al., 2007). The lexicon by Dong et al. (2015) is based on a collection of words from previous lexicons and scales (e.g., Wang et al., 2008). To extend the scope and include new words, researchers also collected words by themselves. Loughran and McDonald (2011), for instance, collected words in financial reports to make their lexicon suitable for detecting emotions in financial markets.

There are two common criteria for selecting words. First, words with low frequency should be excluded. Loughran and McDonald (2011), for instance, deleted the words with frequency of less than 100 in each financial report. Dong et al. (2015) used Weibo’s search engine to exclude low-frequency words. Second, non-emotional words are excluded. For example, Bollen et al. (2011) analyzed word co-occurrences and only kept words related to the terms in emotion scales. For Chinese words, Dong et al. (2015) also suggested that words having the same characters should be deleted. For instance, if “悲苦” (sadness and suffering) is included, “悲苦交加” (mixed feeling of sadness and suffering) is excluded due to the overlap of “悲苦” (sadness and suffering). This strategy may help to prevent the repeated calculation of word frequency (Dong et al., 2015).

### Judging the Emotionality of Words

It is common for sentiment lexicons to output the emotionality of texts by counting the frequency of positive and negative words (Calvo and Kim, 2013). Some lexicons also had ratings of emotions for each word (e.g., Dong, 2014) to improve the accuracy. Thus, it is necessary to determine the emotionality of words in the lexicon. First, a model of emotions should be chosen as the basic rules for the judgment. One approach is to label words as different categories of emotions (Calvo and Kim, 2013), which is usually based on a categorical model of emotions (e.g., happiness, sadness, anger, surprise, disgust, and fear; Ekman and Friesen, 1971). An alternative approach is to focus on the fundamental dimensions of emotions (Calvo and Kim, 2013). Valence (positive versus negative) and arousal (high arousal versus low arousal) are two well-established dimensions in previous literature (e.g., Russell, 1980; Russell and Barrett, 1999).

Second, the emotionality of words can be judged either by human or by machine learning. For instance, human judges determined if a word can be included in a certain category (e.g., positive and negative emotions) when building the Linguistic Inquiry and Word Count (Pennebaker et al., 2007). Dong et al. (2015) used postgraduate students to determine if a word belongs to a certain emotion category (e.g., happiness, sadness, and anger). Other researchers have linked human judgment to machine learning. Wilson et al. (2005), for example, used manual annotations as learning materials to develop a system that can automatically identify the polarity of phrases.

### Establishing the Reliability of the Sentiment Lexicon

Reliability refers to the stability and consistency of social research measurement. It includes test-retest reliability, internal reliability, and inter-rater reliability (Bryman, 2012). Yet according to Pennebaker et al. (2007), examining the internal reliability of a sentiment lexicon is a tricky business, because “the psychometrics of natural language use are not as pretty as with questionnaires. Once you say something, you generally don’t need to say it again in the same paragraph or essay” (Pennebaker et al., 2007, p.9). It is the same for test-retest reliability and inter-observer reliability, because running programs multiple times or in different computers does not change the results.

Nevertheless, reliability issues might be important for examining the stability and consistency of human judgment on the emotionality of lexicon words. Dong (2014), for instance, examined the correlation among human judgment and their test-retest reliability in determining the valence and arousal of words. Likewise, Wilson et al. (2005) calculated the Kappa value, an indicator of agreement rate, for the two annotators’ judgment in labeling the affective polarity of phrases.

### Establishing the Validity of the Sentiment Lexicon

Validity concerns whether the measure can or cannot capture the construct it measures; it mainly includes construct validity (convergent and divergent validity) and predictive validity (Zeidner et al., 2009; Bryman, 2012).

Construct validity includes convergent and divergent validity. For convergent validity, the emotionality captured by a sentiment lexicon (or its words) should be related to that captured by other sentiment lexicons or other valid measures (e.g., human judgment). Dong (2014), for instance, tested the convergent validity of her lexicon by examining the correlations between her ratings with those by Wang et al. (2008) among the same words. A further approach is to link the lexicon output to human judgment. Pennebaker et al. (2007), for instance, tested the relationship between the result generated by the Linguistic Inquiry and Word Count and human judgment among a sample of essays. We did not find much evidence for the divergent validity of sentiment lexicons. But according to emotion theories (e.g., Ekman and Friesen, 1971; Russell, 1980), it is plausible to suggest that the categories or dimensions captured by the lexicon (or its words) should not be highly inter-correlated with each other.

For predictive validity, the emotionality captured by sentiment lexicons should be related to real-life outcomes or behaviors. Some researchers linked word usage to personality traits (Pennebaker and King, 1999; Schwartz et al., 2013; Cutler and Kulis, 2018). For instance, Extraversion, as a trait of sociability and positive emotionality (McCrae and John, 1992), is found to be associated with more use of positive emotional words and less use of negative words (Pennebaker and King, 1999; Yarkoni, 2010). People with high Neuroticism, on the other hand, use more negative emotional words such as “stress” and “depression” (Schwartz et al., 2013; Cutler and Kulis, 2018). Other studies used the output of sentiment lexicons to predict stock market changes (Bollen et al., 2011) and sales outcomes (e.g., book sales; Hu et al., 2014).

### Summary and the Development of CSLI in the Current Study

The development of sentiment lexicons may include four main steps: collecting and selecting words; judging the emotionality of words; establishing the reliability of lexicon; and examining the validity of the lexicon.

For collecting words, previous sentiment lexicons are usually based on dictionary words, previous lexicons, and items in emotion scales (e.g., Pennebaker et al., 2007; Dong et al., 2015). This was also the approach adopted by the current study. We further included a lexicon of internet words in order to better capture the content in online social media. For the selection of words, we excluded low frequent and non-emotional words as common practices in previous literature (e.g., Bollen et al., 2011; Loughran and McDonald, 2011). But unlike Dong et al. (2015), we still kept words with repeated characters. One reason is that many Chinese words include similar characters, such as “快乐” (happy) and “愉快” (happy). Deleting those words may restrict the word coverage of the lexicon. Another reason is that repeated words such as “高兴” (happy) and “不高兴” (unhappy) have opposite meanings; deleting either one may change or even reverse the output of the lexicon. But we are aware of the limitation of using words with repeated characters (e.g., repeated calculation of word frequency), which might be addressed in studies using phrase analysis (e.g., Wilson et al., 2005).

For judging the emotionality of words, previous literature follows either a dimensional or a categorical model of emotions (e.g., Calvo and Kim, 2013). We chose the dimensional model for two reasons. First, the dimensional model, particularly the dimensions of valence and arousal, has been well defined and solidly established in previous literature (Russell, 1980; Russell and Barrett, 1999; Posner et al., 2005). Second, the dimensional model may be more easily operationalized (e.g., letting people judge the valence or the arousal of a particular word), which may be suitable for the initial development of a sentiment lexicon. Accordingly, we applied the model by Russell (1980) in the current lexicon, and included both dimensions of valence and arousal. We also used human judges to determine the emotionality of each word in the lexicon.

Regarding reliability considerations, we followed the suggestions by Pennebaker et al. (2007), and focused on the stability and consistency of human judgment. In particular, we examined test-retest reliability, internal reliability, and inter-rater reliability. Similar to Dong (2014), inter-rater reliability was observed by correlating the results among different judges.

Regarding validity issues, we explored the initial convergent validity by comparing the emotionality of same words in both our lexicon and that by Dong (2014). The convergent validity of the lexicon was further tested by comparing its results with human judgment in a sample of Weibo comments. Finally, following Pennebaker and King (1999), the predictive validity of the lexicon was examined by linking its Weibo results to the personality traits among a sample of undergraduates.

To serve the research purposes, we conducted three studies. In Study 1, we developed CSLI and examined its initial reliability and validity. Study 2 examined the convergent validity of the lexicon by comparing its output with human judgment. Finally, Study 3 addressed the predictive validity of the lexicon by linking its output from a sample of university students’ Weibo postings to their personality traits.

## Study 1

The purpose of Study 1 was to develop CSLI, test its initial reliability and validity. We first collected and selected the words for the lexicon mainly based on previous dictionaries and lexicons. Then we used human judges to determine the emotionality of the words. The reliabilities of human judgment were reported. Finally, the initial validity of the lexicon was examined by comparing its ratings with those by Dong (2014).

### The Collection of Words

We collected initial words from three major sources: (1) dictionaries, (2) previous Chinese sentiment lexicons, and (3) self collection. For the first source, we included the Modern Chinese Corpus (Jin et al., 2005) with words and participles counted more than 50 times in the corpus. We also included the SogouW internet lexicon (Sogou Labs, 2006) with words covering 95% of the total word frequency in the lexicon. Using the internet lexicon may facilitate the capturing of texts in online social media. For the second source, we included the HowNet sentiment (Dong and Dong, 1999), the National Taiwan University Sentiment Dictionary (Ku et al., 2006), the Chinese Affective Lexicon Ontology (Xu L. et al., 2008), the Microblog Basic Emotion Lexicon (Dong, 2014) and the Emotional Dimension Lexicon (Dong et al., 2015). Finally, to enrich the word pool, we also collected words in Weibo by ourselves. In total, 102,270 words were included in the initial collection.

We excluded non-emotional and low-frequent words, and those with uncertain meanings by two rounds of screening. A total of 12 judges were recruited for the tasks. They are divided into four groups with each responsible for 1/4 of the words. The judges were undergraduate students majoring in social work (9 women and 3 men); each of them was given 500 CNY (approximately 100 US dollars). In the first round, Each word was rated by three judge as 0 “Non-emotional,” 1 “Emotional,” and 2 “Uncertain.” The word was included if two of the judges thought it was emotional. This has resulted in 63,492 words. In the second round, judges were required to select the words commonly used in online social media. Each word was rated as 0 “Never/rare,” 1 “Sometimes,” and 2 “Frequently.” The word was included if two of the judges thought it was a frequently used word. This has resulted in a total of 14,217 words.

Finally, repeated words were deleted. All the remaining words were then reviewed by the first author of this paper according to the criteria used in the two-round screening. A total of 7,143 words were finally kept in the lexicon for further analysis.

### Judging the Emotionality of Word

We followed the dimensional model by Russell (1980) and included the dimensions of both valence and arousal in CSLI. The dimension of valence was defined as pleasure-displeasure (Russell, 1980; Russell and Barrett, 1999). Typical pleasure emotions include happy and contented; typical displeasure emotions include upset and sad (Russell, 1980; Russell and Barrett, 1999). The dimension of arousal was defined as the sense of energy or activation of physiological state (Russell, 1980; Russell and Barrett, 1999). Typical high-arousal emotions include tense and alert; typical low-arousal emotions include fatigued and calm (Russell, 1980; Russell and Barrett, 1999).

We used human judgment to measure the emotionality expressed by each word in the lexicon. Twenty judges were recruited, including 15 undergraduates majoring in Chinese and 5 undergraduates majoring in social work (15 women and 5 men). Each judge was given 500 CNY (approximately 100 US dollars). Following the dimensional model, each judge provided their ratings on the valence and arousal of the word in the lexicon independently. In accordance with Dong (2014), their responses were recorded on a nine-point Likert scale (from −4 to 4 for valence; from 0 to 8 for arousal). For words with conflicting emotions, judges are required to give an overall rating (i.e., rating the valence/arousal by taking into accounts all the emotions the word may represent). For the rating of valence, the instruction was as follows:

*When most people used the word in online social media (e.g., Weibo or WeChat), how much valence do you think the people are expressing? Valence means the pleasure-displeasure or positive-negative of the word. Please rate the word on a scale from* −*4 to 4. The higher the score, the more pleasure of the word; the lower the score, the more displeasure of the word. For instance, the valence of the word* “开心” *(happy) can be 4; the valence of the word* “‘ 悲伤” *(sad) can be* −*4. For words with conflicting emotions, please give an overall rating by taking into accounts all the emotions they may represent.*

For the rating of arousal, the instruction was as follows:

When most people used the word in online social media (e.g., Weibo or WeChat), how much arousal do you think the people are expressing? Arousal means the degree of energy or physiological activation expressed by the word. Please rate the word on a scale from 0 to 8. The higher the score, the more energetic or activated of the word; the lower the score, the less energetic or activated of the word. For instance, the arousal of the word “紧张” (tense) can be 8; the arousal of the word “平静” (calm) can be 0. For words with conflicting emotions, please give an overall rating by taking into accounts all the emotions they may represent.

### Testing the Reliability of the Lexicon

Following the advice by Pennebaker et al. (2007), we focused on the reliability of human judgment in determining the emotionality of words in CSLI. For each of the judges, we examined their internal reliability, test-retest reliability, and inter-rater consistency.

For the internal reliability, we randomly included 100 repeated words in the lexicon. Reliability coefficients were computed by the Spearman-Brown formula. The results are shown in Table 1. The mean of the coefficients for valence was 0.85, suggesting that the judges had a high internal consistency in rating the valence of the words. Only Judge 2 and 3 had a coefficient below 0.60. The mean of the coefficient for arousal was 0.49. A close look at the responses of the judges suggests that nearly half of the judges had a coefficient below 0.60.

**TABLE 1:** Internal reliabilities, test-retest reliabilities, and correlations among human judges in rating the valence and arousal in CSLI.

	**J1**	**J2**	**J3**	**J4**	**J5**	**J6**	**J7**	**J8**	**J9**	**J10**	**J11**	**J12**	**J13**	**J14**	**J15**	**J16**	**J17**	**J18**	**J19**	**J20**	**IRV**	**IRA**	**TRV**	**TRA**
J1	–	0.10	0.09	0.28	0.14	0.11	0.14	0.19	0.17	0.21	0.08	0.16	0.26	0.15	0.26	0.26	0.14	0.16	0.27	0.07	0.92	0.68	0.88	0.46
J2	0.46	–	0.07	0.14	0.06	0.05	0.07	0.10	0.09	0.14	0.02	0.05	0.11	0.07	0.11	0.11	0.07	0.03	0.12	0.06	0.47	0.22	0.34	0.25
J3	0.54	0.31	–	0.08	0.10	0.11	0.06	0.09	0.08	0.11	–0.06	0.09	0.07	0.04	0.07	0.08	0.08	0.01	0.10	0.09	0.56	0.21	0.56	–0.04
J4	0.82	0.45	0.57	–	0.10	0.12	0.18	0.33	0.21	0.41	0.01	0.22	0.29	0.14	0.44	0.40	0.11	0.18	0.90	0.14	0.97	0.60	0.94	0.64
J5	0.81	0.45	0.55	0.82	–	0.13	0.08	0.15	0.15	0.11	0.01	0.28	0.21	0.06	0.11	0.13	0.16	0.13	0.10	0.07	0.79	0.11	0.82	0.46
J6	0.70	0.36	0.47	0.72	0.76	–	0.16	0.21	0.16	0.13	–0.14	0.14	0.14	0.03	0.16	0.16	0.06	0.04	0.18	0.15	0.87	0.60	0.85	0.72
J7	0.58	0.29	0.44	0.60	0.59	0.53	–	0.23	0.24	0.26	–0.15	0.21	0.26	0.09	0.28	0.26	0.07	0.03	0.19	0.14	0.67	0.34	0.70	0.13
J8	0.82	0.45	0.55	0.83	0.84	0.74	0.58	–	0.31	0.41	–0.19	0.21	0.30	0.12	0.35	0.41	0.07	0.12	0.36	0.16	0.97	0.74	0.93	0.60
J9	0.80	0.42	0.56	0.81	0.82	0.71	0.59	0.81	–	0.33	–0.21	0.28	0.39	0.17	0.30	0.35	0.09	0.11	0.21	0.14	0.90	0.61	0.86	0.78
J10	0.78	0.43	0.55	0.81	0.82	0.74	0.59	0.80	0.79	–	–0.24	0.16	0.30	0.18	0.42	0.49	0.01	0.14	0.43	0.23	0.94	0.67	0.88	0.66
J11	0.81	0.44	0.57	0.81	0.80	0.67	0.58	0.81	0.81	0.77	–	0.01	0.07	–0.01	–0.01	–0.10	0.35	0.21	–0.02	–0.16	0.93	0.46	0.83	0.38
J12	0.78	0.42	0.54	0.78	0.78	0.68	0.57	0.78	0.78	0.77	0.78	–	0.39	0.14	0.23	0.20	0.26	0.13	0.22	0.01	0.85	0.67	0.66	0.53
J13	0.82	0.44	0.56	0.82	0.82	0.73	0.59	0.83	0.81	0.81	0.80	0.79	–	0.23	0.47	0.41	0.20	0.28	0.27	0.09	0.95	0.86	0.78	0.76
J14	0.64	0.39	0.47	0.65	0.66	0.57	0.46	0.66	0.63	0.65	0.64	0.63	0.66	–	0.18	0.18	0.09	0.06	0.13	0.05	0.63	–0.05	0.83	0.59
J15	0.80	0.44	0.56	0.83	0.80	0.72	0.58	0.81	0.80	0.81	0.80	0.77	0.81	0.65	–	0.55	0.10	0.23	0.46	0.19	0.99	0.99	0.83	0.69
J16	0.81	0.46	0.57	0.83	0.85	0.73	0.59	0.84	0.81	0.82	0.81	0.78	0.83	0.68	0.83	–	0.06	0.22	0.42	0.19	0.94	0.78	0.31	0.52
J17	0.78	0.38	0.54	0.79	0.80	0.72	0.57	0.80	0.81	0.78	0.78	0.77	0.80	0.61	0.78	0.79	–	–0.02	0.11	–0.05	0.93	0.22	0.90	0.53
J18	0.79	0.45	0.56	0.81	0.82	0.71	0.58	0.81	0.81	0.80	0.79	0.78	0.81	0.64	0.78	0.82	0.78	–	0.17	0.06	0.95	0.54	0.88	0.43
J19	0.82	0.45	0.57	0.97	0.82	0.73	0.60	0.82	0.81	0.81	0.81	0.78	0.82	0.65	0.83	0.83	0.79	0.80	–	0.16	0.96	0.51	0.65	0.64
J20	0.74	0.40	0.50	0.73	0.73	0.63	0.52	0.74	0.71	0.70	0.73	0.71	0.72	0.58	0.73	0.73	0.71	0.71	0.73	–	0.87	0.00	0.79	–0.26

*The below half of the matrix are the correlations on valence; the up half are the correlations on arousal. Internal and test-retest reliabilities were based on the ratings of 100 repeated words. Inter-rater correlations were based on all the 7,143 words in CSLI. IRV, Internal reliability for valence; IRA, Internal reliability for arousal; TRV, Test-retest reliability for valence; TRA, Test-retest reliability for arousal. All the correlation coefficients more than 0.23 had a significant level of 0.05.*

For the test-retest reliability, we conducted a training session 1 week before the judges rated the whole lexicon. In the training session, the judges were asked to finish the rating of 100 randomly selected words from the lexicon. Their results were then compared with their ratings of the same words later (with one-month interval). Reliability coefficient was computed by the Spearman-Brown formula. As shown in Table 1, the mean of the coefficients was 0.76 for valence, suggesting that the ratings of valence were quite stable. Only Judge 2, 3, and 16 had a coefficient below 0.60. The mean of the coefficients was 0.47 for arousal. Nearly half of the judges had a coefficient below 0.60.

Finally, inter-rater consistency was observed by calculating the correlations among the responses of the judges. As shown in Table 1, the mean of correlation coefficients for valence was 0.69, suggesting that the judges had a high degree of agreement in rating the valence of the words. Yet the mean of correlation coefficients was 0.16 for arousal, suggesting that there was less agreement on the arousal of the words among the judges.

To ensure the quality of judgment, we only included the judges who passed all the tests of internal and test-retest reliabilities (i.e., had coefficients above 0.60) for further analysis. This has resulted in seven judges (Judge 4, 6, 8, 9, 10, 13, and 15) being retained. The degree of valence and arousal for each of the words in the lexicon was then calculated by averaging their ratings.

### Testing the Validity of the Lexicon

Following the approach by Dong (2014), we examined the initial convergent validity of the lexicon by comparing the rating of valence and arousal in both Dong (2014) and in our lexicon. There were a total number of 654 same words in both lexicons. The correlations between Dong (2014) and our lexicon were shown in Table 2. The correlation between the dimensions of valence was 0.97 (*p* < 0.001), suggesting that the rating of CSLI had a high agreement with that of Dong (2014). The correlation between the dimensions of arousal was 0.73 (*p* < 0.001), which also indicated a high agreement between the two lexicons. Accordingly, our testing indicated that the initial CSLI had an acceptable level of the convergent validity.

**TABLE 2:** Correlations of CSLI and Dong (2014) (*n* = 654).

	**1**	**2**	**3**
(1) Valence (Dong, 2014)	–		
(2) Valence (CSLI)	0.97^∗∗∗^	–	
(3) Arousal (Dong, 2014)	−0.09^∗^	–0.13^∗∗^	–
(4) Arousal (CSLI)	–0.19^∗∗∗^	–0.21^∗∗∗^	0.73^∗∗∗^

*^∗^*p* < 0.05, ^∗∗^*p* < 0.01, ^∗∗∗^*p* < 0.001.*

We examined the divergent validity of the lexicon by comparing the dimension of valence with that of arousal among the words in the lexicon. According to the model by Russell (1980), valence and arousal were two independent dimensions. As shown in Table 2, our result also suggested that there was only a small correlation (*r* = −0.21, *p* < 0.001) between the two dimensions. In other words, the rating of valence was different from that of arousal among the words in our lexicon, which was in line with the dimensional model.

### Discussion

Following previous literature, we developed CSLI by collecting initial words from three main sources: general and internet dictionaries, existing sentiment lexicons, and self-collection. After two rounds of screening, we excluded non-emotional, low-frequency, and repeated words. The final lexicon had 7,143 words.

The valence and arousal of each word in the lexicon was determined by human judgment. Twenty judges provided their ratings independently. The reliability of the human judgment was examined including their internal consistency, test-retest stability, and inter-rater correlations. Reliability regarding the dimension of valence was generally acceptable. The low consistency in judging the dimension of arousal might be due to the individual differences in judging the arousal of emotional experiences (e.g., interoceptive sensitivity; Barrett et al., 2004). It may also be affected by the quality of the judgment (since some judges had low internal and test-retest reliabilities). As a *post hoc* analysis, we conducted an exploratory factor analysis based on the ratings of the seven judges who had stable judgment and were finally included in calculating the emotionality of the words. The result yielded one factor with an eigenvalue of 2.85 accounting for 40.65% of the variance. In other words, although the judges had a certain level of disagreement in rating the dimension of arousal, a general factor of arousal still existed.

For the construct validity, we computed the correlation between our ratings and those by Dong (2014) among 654 repeated words. The results suggested that the two lexicons had a high convergence on the dimensions of valence and arousal, respectively. The two dimensions also had a low correlation. Accordingly, the initial validity of CSLI was established.

## Study 2

The purpose of Study 2 was to further establish the construct validity of CSLI. Following the approach by Pennebaker et al. (2007), we compared human judgment with the results of the lexicon. The sample was 429 aggregated Weibo comments, exhibiting degrees of emotion about the subject matter involved (Xu T. et al., 2008).

### Method

#### Sample

We collected the Weibo comments in three steps. First, we located a sample of famous Weibo accounts by including a list from the Weibo V influence Summit 2015 (Baidu, 2016), which honored the yearly most influential Weibo authors in 2015. Choosing these accounts helped to ensure that there were sufficient comments for analysis. The list included both organizations and individuals, covering a range of industries (e.g., entertainment, finance, media, medical, sports, technology…) and topics (domestic and international news, cartoons, fashion, jokes, movies, photograph…). We also collected the top 10 “hot” accounts from each of the Weibo categories using the navigation bar on the main page of Weibo. A total of 386 Weibo accounts were finally located. Second, we collected the top 10 Weibo postings by each of those accounts and their related comments. Finally, we screened the posted Weibo and its comments, and only included Weibo postings having more than 500 comments. We also deleted Weibo postings which may not generate emotions (e.g., giving a multiple-choice question, or asking people to give their family name). This resulted in a total of 429 Weibo postings and their comments. We only kept the first 500 comments under each posting to control for the effect of the total number of comments on the results of the lexicon. We also cleaned out the expression of “@” and Weibo ID in each comment. Comments were then aggregated according to each posting, yielding 429 aggregated comments. All the personal and account information were not included in the final data to keep the data anonymous.

#### Measures

##### CSLI

The new lexicon developed in Study 1 was used to capture the valence and arousal of the aggregated comments. The indicator of valence was computed by the weighted average of the valence of each word in the lexicon. In particular, we first summed up the product of the valence of each word multiplied by their frequency in the comments; the sum was then divided by the total frequency of the words. The indicator of arousal was computed using the same method. Weighted average has been frequently used to balance the effect of word frequency in calculating the emotions of texts using sentiment lexicons (e.g., Bollen et al., 2011; Dong, 2014).

##### Human judgment

Three judges were recruited to rate the valence and arousal of the Weibo comments under each Weibo posting. The judges were undergraduates majoring in social work (two women and one man). Each of the students was provided 400 CNY (approximately 80 US dollars). We did not include the same judges from Study 1, because previous rating experiences may influence their judgment on the Weibo comments. Each judge was asked to rate the aggregated Weibo comments independently on a nine-point Likert scale (from −4 to 4 for valence; from 0 to 8 for arousal). The judges were also encouraged to read the original Weibo posting as a reference for their judgment. For the rating of valence, the instruction was as follows:

*Please carefully read the comments under each of the Weibo postings. In all, how much valence do you think the people are expressing through their comments? Valence means the pleasure-displeasure or positive-negative of their comments. Please rate on a scale from* −*4 to 4. The higher the score, the more pleasure in their comments; the lower the score, the more displeasure in their comments.*

For the rating of arousal, the instruction was as follows:

Please carefully read the comments under each of the Weibo posting. In all, how much arousal do you think the people are expressing through their comments? Arousal means the degree of energy or physiological activation of their comments. Please rate on a scale from 0 to 8. The higher the score, the more energetic or activated their comments are; the lower the score, the less energetic or activated their comments are.

The three judges had a high inter-rater consistency in judging the valence of the Weibo comments with an average correlation coefficient (based on Pearson’s *r*) of 0.67. Similar to Study 1, the inter-rater consistency in judging the arousal of the Weibo comments was relatively low with an average correlation coefficient of 0.30, suggesting potential individual differences in perceiving emotional arousal (e.g., Barrett et al., 2004). In order to produce a unified indicator of valence and arousal for each aggregated Weibo comments, we averaged the ratings of the three judges.

#### Procedure

Weibo postings and their comments are open to the public. We built a crawler to capture and aggregate the Weibo comments. To keep the anonymity of data, we cleared all the ID information in the comments. A computer program was then designed to output the valence and arousal of each aggregated comment using CSLI. Human judges were first trained by providing five examples of ratings according to the instructions given. They then completed the whole ratings independently. It took almost 1 week to complete the rating of the Weibo comments.

### Results

Table 3 shows the mean and SD of the human judgment and the output of CSLI, and their inter-correlations. The output of valence by CSLI was positively related to that by human judges (*r* = 0.70, *p* < 0.001), suggesting that the output of the lexicon has a high degree of agreement with the human judgment. Likewise, the rating of arousal by CSLI also positively correlated with the human judgment (*r* = 0.59, *p* < 0.001), indicating that the outcome of the lexicon was consistent with the human judgment. Accordingly, the results suggest that CSLI has convergent validity in determining the valence and arousal of the Weibo comments. It is also interesting to see that the dimensions of valence and arousal were positively correlated in both human judgment (*r* = 0.70, *p* < 0.001) and lexicon outputs (*r* = 0.73, *p* < 0.001), which may imply some co-occurrence of the two dimensions among the commenting words.

**TABLE 3:** Correlations of CSLI and human judgment in Weibo comments (*n* = 429).

	**M**	**SD**	**1**	**2**	**3**
(1) Valence (Human)	1.48	1.47	–		
(2) Valence (CSLI)	0.58	0.32	0.70^∗∗∗^	–	
(3) Arousal (Human)	5.42	0.79	0.70^∗∗∗^	0.53^∗∗∗^	–
(4) Arousal (CSLI)	2.57	0.22	0.63^∗∗∗^	0.73^∗∗∗^	0.59^∗∗∗^

*^∗^*p* < 0.05, ^∗∗^*p* < 0.01, ^∗∗∗^*p* < 0.001.*

### Discussion

Study 2 tested the convergent validity of CSLI by comparing its results with human judgment on the valence and arousal of Weibo comments. The results suggested that the ratings of valence and arousal by the lexicon had positive correlations with the ratings by human judges. Accordingly, the convergent validity of CSLI has been further established. Interestingly, the dimensions of valence and arousal were also positively inter-correlated among the Weibo comments. Because Study 1 showed that the two dimensions were independent among the ratings of words in the lexicon, the result may be due to the linguistic style of the Weibo commenters. In other words, words used in the Weibo comments may tend to be either pleasure and high-arousal or displeasure and low-arousal. By looking at frequency of each word in the comments, we found that many comments included positive and high arousal words such as “ 哈哈” (hahah), “ 爱” (love), and “喜欢” (like).

### A *Post hoc* Replication Study

Because all the three judges in Study 2 were undergraduate students, to replicate and generalize the research findings, we conducted a *post hoc* study by inviting three new judges (two women and one man) to participate in the research. Two of them were postgraduate students majoring in social work; one of them was a fund manager (age 35). They were asked to rate the valence and arousal of the same Weibo comments in Study 2 independently. The mean of their ratings was then compared with the output from CSLI. The results had generally replicated the findings of Study 2. The mean valence from the new human ratings had a positive correlation with the output from CSLI (*r* = 0.75, *p* < 0.001). Likewise, the mean arousal was also positively related to the output from CSLI (*r* = 0.47, *p* < 0.001). Accordingly, the validity of the findings in Study 2 was established. Detail of the *post hoc* study is available from the corresponding author.

## Study 3

The purpose of Study 3 was to further explore the predictive validity of CSLI. Following Pennebaker and King (1999), we linked the results of the lexicon to personality traits. In particular, we captured the valence and arousal in Weibo postings among a sample of undergraduate students and linked the results to their self-report personality traits. As the literature suggested, the traits of Extraversion was related with more use of positive and less use of negative emotion words (Pennebaker and King, 1999; Yarkoni, 2010; Cutler and Kulis, 2018), so were Agreeable (as the trait of warmth and caring) and Conscientiousness (as the trait of diligent and strong will) (Pennebaker and King, 1999; Schwartz et al., 2013). Furthermore, since Extraverts should be energetic and highly sensation-seeking (McCrae and John, 1992), they frequently used high-arousal emotional words such as “excited” and “love” in the online social media (Schwartz et al., 2013; Cutler and Kulis, 2018). In contrast, Neuroticism was related to more use of negative and less use of positive emotional words (Pennebaker and King, 1999; Schwartz et al., 2013). Accordingly, we made the following hypotheses:

1. *Hypothesis 1*: Valence captured by CSLI in students’ Weibo postings was positively related to their Extraversion.
2. *Hypothesis 2*: Arousal captured by CSLI in students’ Weibo postings was positively related to their Extraversion.
3. *Hypothesis 3*: Valence captured by CSLI in students’ Weibo postings was positively related to their Agreeableness.
4. *Hypothesis 4*: Valence captured by CSLI in students’ Weibo postings was positively related to their Conscientiousness.
5. *Hypothesis 5*: Valence captured by CSLI in students’ Weibo postings was negatively related to their Neuroticism.

### Method

#### Sample

A sample of 71 undergraduate students were initially recruited. They were informed about the research purpose, and were voluntary to participate in the research by providing their Weibo accounts. As an incentive to participate, each of the participants was provided 100 CNY (approximately 20 US dollars). In order to ensure that there were enough texts for analysis, we excluded participants whose Weibo account had been used for less than 1 year and had less than 50 original Weibo postings. The final sample included 52 students (12 men and 40 women). Their average age was 21.35 years (SD = 1.67). To control for the effect of the number of postings on the results, only the first 500 Weibo postings by each participant were collected. Retweeted postings without original content were not included in the research because retweeted content may not reflect the writing style and personal traits of the individual. The expression of “@” and its related ID were also cleaned out. The postings by each participant were then aggregated at the individual level for further analysis. All the personal and account information were deleted to protect personal privacy and keep the final dataset anonymous.

#### Measures

##### CSLI

We captured the valence and arousal of the Weibo postings using the same method in Study 2. Weighted averages of the lexicon words were computed to produce the results of valence and arousal for each Weibo account.

##### Personality traits

We measured the traits of the participants using a 10-item version of the Big Five Inventory (Rammstedt and John, 2007). There are two items for each of the five traits in the inventory. For Extraversion, the items are “is reserved” (reversed scoring) and “is outgoing, sociable”; for Agreeableness, the items are “is generally trusting” and “tends to find fault with others” (reversed scoring); for Conscientiousness, the items are “tends to be lazy” (reversed scoring) and “does a thorough job”; for Neuroticism, the items are “is relaxed, handles stress well” (reversed scoring) and “gets nervous easily”; for Openness to experiences, the items are “has few artistic interests” (reversed scoring) and “has an active imagination.” Participants rated the items on a five-point scale from 1 = strongly disagree to 5 = strongly agree. The score for each trait was computed by averaging the responses of the two items. The inventory is easy to use, and has good reliability and validity in previous studies (e.g., Rammstedt and John, 2007). A Chinese translation of the inventory was provided by D. Cai (personal communication, May 27, 2016). In the current study, the correlations of two items for each trait was −0.50 (*p* < 0.001, for Extraversion), −0.02 (*p* > 0.05, for Agreeableness), −0.06 (*p* > 0.05, for Conscientiousness), −0.37 (*p* < 0.01, for Neuroticism). −0.24 (*p* > 0.05, for Openness to experiences).

##### Control variables

We captured the gender (1 = male, 2 = female) and age (years) of the participants. We also controlled the number of total Weibo postings (including both original and retweeted postings), followers, and following in their Weibo accounts.

#### Procedure

We posted an advertisement in the campus bulletin board to recruit participants. Potential participants can use their Weibo app to scan the QR code in the advertisement to read the full participation information statement. Those who agreed to participate were redirected to the online survey. They completed the personality measure and provided the name of their Weibo accounts for data collection purposes. Those who gave a valid Weibo account were contacted via Weibo and provided with the participation incentives. We adjusted the crawler in Study 2 to capture and aggregate the Weibo postings by each account. We also calculated the valence and arousal of the Weibo postings using the same program in Study 2. The final data was kept anonymous by deleting all the personal and account information.

### Results

The participants posted an average of 2313.71 postings (SD = 4079.85), had an average of 595 followers (SD = 1839.85), and followed an average of 320.77 (SD = 247.53) other Weibo accounts. We noticed that two participants were also influential Weibo authors; each of them had more than 10,000 postings in total (including both original and retweeted postings).

Table 4 provides the description and correlations of the research variables. To further test the hypotheses, we conducted partial correlations by ruling out the effect of control variables. The result of partial correlations suggested that the valence of Weibo postings was positively related to the Extraversion of the participants (*r* = 0.37, *p* < 0.05), indicating that those with high Extraversion used more positive and less negative words in their Weibo postings. Accordingly, *Hypothesis 1* was supported. The arousal of Weibo postings was also positively related to the Extraversion of the participants (*r* = 0.39, *p* < 0.01), indicating that participants with high Extraversion also tended to use high-arousal words in their Weibo postings. Accordingly, *Hypothesis 2* was supported. Yet the correlation between Agreeableness and valence was not significant (*r* = 0.01, *p* = 0.94). *Hypothesis 3* was thus not supported. Weibo valence was positively related to the Conscientiousness of the participants (*r* = 0.31, *p* < 0.05), indicating that participants with high Conscientiousness used more positive and less negative words in their Weibo postings. Accordingly, *Hypothesis 4* was supported. Finally, valence in Weibo postings was negatively related to Neuroticism (*r* = −0.35, *p* < 0.05), suggesting that participants with high Neuroticism used less positive and more negative words in their Weibo postings. Accordingly, *Hypothesis 5* was supported. Overall, the output of CSLI based on undergraduate students’ Weibo posting was related to their personality traits. The predictive validity of CSLI has been largely supported.

**TABLE 4:** Correlations of CSLI and personality traits in Weibo postings by undergraduate students (*n* = 52).

	**M**	**SD**	**1**	**2**	**3**	**4**	**5**	**6**	**7**	**8**	**9**	**10**	**11**
(1) Gender	1.77	0.43											
(2) Age	21.35	1.67	–0.11										
(3) No. of postings	2313.71	4079.85	0.09	0.18									
(4) No. of following	320.77	247.53	0.04	0.05	0.30^∗^								
(5) No. of followers	595.40	1839.85	–0.23	0.24	0.49^∗∗∗^	0.21							
(6) Extraversion	3.26	0.94	–0.02	–0.08	0.14	0.10	0.26						
(7) Agreeableness	3.57	0.70	0.15	–0.07	−0.32^∗^	–0.12	–0.12	0.16					
(8) Conscientiousness	3.01	0.70	0.01	–0.07	–0.01	0.00	–0.05	0.03	0.22				
(9) Neuroticism	3.40	0.79	–0.07	–0.02	0.11	0.05	0.09	−0.32^∗^	0.12	0.00			
(10) Openness	3.71	0.81	0.14	0.08	0.37^∗∗^	0.19	0.22	0.13	–0.03	0.05	0.08		
(11) Valence (CSLI)	0.51	0.23	0.02	–0.03	–0.15	0.17	–0.20	0.28^∗^	0.04	0.30^∗^	−0.35^∗^	–0.13	
(12) Arousal (CSLI)	2.59	0.27	0.04	–0.06	0.25	0.09	0.09	0.40^∗∗^	0.14	0.23	–0.04	–0.04	0.37^∗∗^

*^∗^*p* < 0.05, ^∗∗^*p* < 0.01, ^∗∗∗^*p* < 0.001.*

### Discussion

Study 3 further tested the predictive validity of CSLI by linking its outcomes of valence and arousal in Weibo postings to personal traits. The results generally supported the research hypotheses: valence and arousal of Weibo postings were positively related to Extraversion; valence was also positively related to Conscientiousness, and negatively related to Neuroticism. Accordingly, the predictive validity of the lexicon was established. Yet the correlation between Weibo valence and Agreeableness was not significant. A possible reason may be that although people with high Agreeableness tend to use more positive words in their writings, they may also use negative words such as “sorry,” “sigh,” and “worry” (Iacobelli et al., 2011; Cutler and Kulis, 2018). In other words, people with high Agreeableness may also be empathetic to others’ negative feelings (Tobin et al., 2000).

### A *Post hoc* Replication Study

In Study 3, the reliability of the personality measure might be questionable due to the low correlations among its items, which may also affect the replicability of the research findings. To further verify the findings, we conducted a *post hoc* study by applying the same research design and procedure of Study 3. We replaced the 10-item Big Five Inventory (Rammstedt and John, 2007) with a Chinese version of the 44-item Big Five Inventory (Carciofo et al., 2016). The sample included 65 individuals (13 men and 52 women). Their mean age was 22.83 years (SD = 1.53). 29 of them were participants from Study 3 (who redid the personality measure), 36 of them were newly recruited participants (who were mainly undergraduate and postgraduate students majoring in sociology or social work). All the personality dimensions had a Cronbach’s α more than 0.70. More importantly, the results had generally replicated the findings of Study 3. After ruling out the effect of control variables, the valence of Weibo posting (generated by CSLI) was positively related to Extraversion (*r* = 0.43, *p* < 0.001) and Conscientiousness (*r* = 0.26, *p* < 0.05), and negatively related to Neuroticism (*r* = −0.22, *p* < 0.1). The arousal of Weibo positing was also positively related to Extraversion (*r* = 0.30, *p* < 0.05). The partial correlation between the valence of Weibo posting and Agreeableness was not significant (*r* = −0.00, *p* = 0.98). Therefore, the validity of the research findings in Study 3 was confirmed. Detail of the *post hoc* study is available from the corresponding author.

## Conclusion

The main aim of this research is to build a reliable and valid Chinese sentiment lexicon suitable for capturing the valence and arousal in online social media texts (e.g., Weibo and WeChat). To serve this purpose, we conducted three studies. In Study 1, we built up CSLI by collecting words from previous dictionaries and lexicons, and rating the valence and arousal of the words using human judges. The initial reliability and validity of CSLI were established by examining the consistency of the human judgment and its correlation with the lexicon by Dong (2014). Study 2 further explored the construct validity of CSLI by comparing its result with human judgment in aggregated Weibo comments. Similar to Pennebaker et al. (2007), we found a positive connection between the results by CSLI and those by human judgment. Finally, we tested the predictive validity of CSLI in Study 3 by linking its outputs from Weibo postings to self-rated personality traits among a sample of undergraduate students. In line with previous literature (Pennebaker and King, 1999; Yarkoni, 2010; Schwartz et al., 2013), the results confirmed the relationship between valence and arousal captured by CSLI and self-report personality traits. We also conducted two *post hoc* replication studies to verify the findings of Study 2 and 3. Overall, the reliability and validity of CSLI were supported in the current study.

This research has three contributions to the literature. First, we reviewed the major steps and techniques in building up a sentiment lexicon in previous literature, which might be useful for future studies aiming to develop sentiment lexicons in other languages. Second, following the literature, we developed CSLI, a Chinese sentiment lexicon, which was reliable and valid for capturing the valence and arousal in online social media texts. The lexicon may benefit and further extend the research of sentiment analysis using Chinese texts. Finally, as we examined the predictive validity of CSLI, we replicated the findings using English lexicons (e.g., Pennebaker and King, 1999; Schwartz et al., 2013), and confirmed the links between online social media texts and personality traits among their posters.

The research has several practical implications for CSLI as a means of promoting human well-being. First, the results of CSLI can be used to reflect public emotions toward certain events or policies, thus facilitating the decision-making process. For example, Xu T. et al. (2008) captured the public emotions during emergencies to provide more information for decision-making in emergency response. Second, a self-reflection tool can be designed based on CSLI to increase people’s self-awareness about their emotions during a certain period, thus promoting their self-management and well-being in turn (Calvo and Peters, 2017). Third, since the results of CSLI were linked with personality traits, people may use the results as references for personal improvement and maintaining positive self-image in online social media.

The research is not without limitations. First, since the research is exploratory in nature, we have only included a dimensional model in CSLI. In future studies, it might be necessary to further include a categorical model (e.g., Ekman and Friesen, 1971) in the lexicon to provide more information regarding the specific emotions in online social media. Second, although we collected “internet” words using the SogouW internet lexicon (Sogou Labs, 2006) and by our own means, recently invented words have not been included in the lexicon. Hence, it is important for CSLI to update its words regularly in the future. Finally, due to time and cost, we only used a relatively small sample of university students to test the predictive validity of CSLI. Its predictive effect needs to be further examined in future research. The use of CSLI may also be limited to the Chinese sociocultural context due to the different languages and cultures in other sociocultural contexts.

In conclusion, based on a review of the main steps and techniques in building sentiment lexicons, we developed CSLI, a Chinese sentiment lexicon suitable for capturing emotions in online social media texts (e.g., Weibo and WeChat). The initial reliability and validity of CSLI were established in three studies. The results of CSLI were comparable to human judgment, and were related to personality traits. Future studies can use CSLI as a tool to further explore the emotions in Chinese online social media texts.

## Data Availability Statement

The datasets for this manuscript are not publicly available because they contain Weibo postings by the research participants, and the detail of the lexicon built by the authors. It is our responsibility to protect the personal privacy of the participants, and the intellectual property of the authors. Requests to access the datasets should be directed to the corresponding author.

## Ethics Statement

This study was carried out in accordance with the Declaration of Helsinki and the recommendations of the ethical standards for institutional research with human subjects. A passive consent procedure was applied by informing the participants about the nature of the research. The research protocol was approved by the Research Ethics Committee, Shanghai Normal University.

## Author Contributions

J-LZ started and supervised the whole research project, analyzed the data, and wrote the manuscript. M-ZL designed and conducted Study 3. JY reviewed and revised the manuscript. G-HQ built the crawler program.

## Conflict of Interest

The authors declare that the research was conducted in the absence of any commercial or financial relationships that could be construed as a potential conflict of interest.
